# Phytochemical Characterization of *Dillenia indica* L. Bark by Paper Spray Ionization-Mass Spectrometry and Evaluation of Its Antioxidant Potential Against t-BHP-Induced Oxidative Stress in RAW 264.7 Cells

**DOI:** 10.3390/antiox9111099

**Published:** 2020-11-09

**Authors:** Md Badrul Alam, Arif Ahmed, Syful Islam, Hee-Jeong Choi, Md Abdul Motin, Sunghwan Kim, Sang-Han Lee

**Affiliations:** 1 Department of Food Science and Biotechnology, Graduate School, Kyungpook National University, Daegu 41566, Korea; mbalam@knu.ac.kr (M.B.A.); choi930302@gmail.com (H.-J.C.); 2 Food and Bio-Industry Research Institute, Inner Beauty/Antiaging Center, Kyungpook National University, Daegu 41566, Korea; 3 Department of Chemistry, Kyungpook National University, Daegu 41566, Korea; arifahmed83@gmail.com (A.A.); msi412@yahoo.com (S.I.); 4 Department of Chemistry, University of California, Riverside, CA 92521, USA; mdabdulm@ucr.edu; 5 Mass Spectrometry Converging Research Center and Green-Nano Materials Research Center, Daegu 41566, Korea; 6 knu BnC, Daegu 41566, Korea

**Keywords:** antioxidant, *Dillenia indica*, heme oxygenase 1 (HO-1), nuclear factor erythroid 2-related factor 2 (Nrf2), RAW 264.7 cells

## Abstract

The antioxidant effects of the ethyl acetate fraction of *Dillenia indica* bark (DIBEt) and the underlying mechanisms were investigated in *tert*-butyl hydroperoxide (t-BHP)-stimulated oxidative stress in RAW 264.7 cells. Paper spray ionization-mass spectroscopy with positive-ion mode tentatively revealed 27 secondary metabolites in *D. indica* bark extract; predominant among them were alkaloids, phenolic acids, and flavonoids. A new triterpenoid (nutriacholic acid) was confirmed in DIBEt for the first time. DIBEt had strong free radical-scavenging capabilities and was also able to reduce t-BHP-induced cellular reactive oxygen species (ROS) generation in RAW 264.7 cells. DIBEt was found to prevent oxidative stress by boosting the levels of heme oxygenase-1 (HO-1) through the up-regulation of nuclear factor erythroid 2-related factor 2 (Nrf2) via the regulation of extracellular signal-regulated kinase (ERK) phosphorylation in RAW 264.7 cells. These results support the potential of DIBEt for defense against oxidative stress-stimulated diseases.

## 1. Introduction

Among the various signaling molecules, reactive oxygen species (ROS) and reactive nitrogen species (RNS) play critical roles in maintaining cellular homeostasis. Redox imbalance precisely participates in the pathogenesis and pathophysiology of numerous chronic diseases [1]. However, macrophage cells serve as the first line of defense in infected cells, and activated macrophages are a major source of ROS and RNS triggers epigenetic modifications, leading to the pathogenesis of chronic diseases [2]. Thus, activated macrophage models can identify the active components for functional diet development through a multiple-target strategy [2]. Natural medicinal products have been exploited in medical practice for centuries. Phytochemicals with inherent antioxidant potential orchestrate innumerable cellular defensive signaling cascades directly or indirectly and might have remedial applications for oxidative stress-induced disorders [3]. Thus, it is essential to understand and validate the bioactivities of natural compounds and their molecular mechanisms to form concrete scientific evidence for their clinical use and effectiveness and to meet regulatory challenges.

Nuclear factor erythroid 2-related factor 2 (Nrf2) activation triggers the induction of various detoxifying and antioxidant enzymes, such as heme oxygenase-1 (HO-1) and NAD(P)H quinone oxidoreductase 1 (NQO1) [4]. In the resting state, cytosolic Kelch-like ECH-associated protein 1 (Keap1) causes the degradation of Nrf2 through the ubiquitin-proteasome system. During stress conditions or xenobiotic challenge, the reactive cysteine residue of Keap1 is modified, causing the conformational change of Keap1 structure that prevents Nrf2 degradation, which is then free to translocate to the nucleus and bind to antioxidant-related elements (AREs) in the promoter regions of antioxidant and cytoprotective genes [4]. Furthermore, the activation of mitogen-activated protein kinase (MAPK), phosphatidylinositol 3-kinase/Akt (PI3K/AKT), and protein kinase C (PKC) also boosts Nrf2 nuclear translocation [5].

Instrumental analytical techniques, such as high-performance liquid chromatography (HPLC) and gas chromatography (GC) coupled with mass spectrometry (MS), have been applied to qualitatively and quantitatively explore the secondary metabolites of medicinal plants or foods. Although these techniques are very accurate and precise, they are very time-consuming and require laborious sample preparation and high costs. In contrast, paper spray ionization-MS (PSI-MS) requires minimal sample preparation time, boosts the ionization of compounds under mild experimental conditions, and furnishes ultrafast examinations of complex matrices at low cost [6]. Thus, PSI-MS has been widely accepted in resveratrol evaluation in red wine [7], chemical composition and fraud verification of whiskey and beer [8], medicines, pesticide analysis in fruits and vegetables, and food additives and their byproducts [9].

*Dillenia indica* (family Dilleniaecae) is commonly known as elephant apple. The pulp of the fruit is applied on the scalp to cure dandruff and hair loss, and the sepal has been used to treat stomach disorders since ancient times [10]. Evidence suggested that *D. indica* possesses various medicinal properties, such as anticancer [11], antimicrobial, antioxidant [12], analgesic, anti-inflammatory [13], and antidiabetic and its associated complications, such as hyperlipidemia [14], diabetic nephropathy, and neuropathy. However, little information is available on the chemical composition of *D. indica* bark (DIB). Therefore, this study aimed to provide further information on the chemical composition of the ethyl acetate fraction of DIB (DIBEt) through the determination of total phenolic and total flavonoid contents and in-vitro antioxidant capacity. The bioactive components of DIBEt were screened using PSI-MS. Furthermore, the focus was on the regulatory role of DIBEt on the expression of antioxidant enzymes in RAW 264.7 cells and the underlying mechanisms.

## 2. Materials and Methods

### 2.1. Plant Materials and Extraction

DIB was collected from Jahangirnagar University, Bangladesh, in August 2018 and taxonomically identified by the National Herbarium of Bangladesh (voucher specimen no. 49403) and retained in the laboratory for future reference. Dried and coarsely powdered barks (100 g) were extracted by shaking with ethanol at 60 °C for 12 h (three times) and dried in a rotary vacuum evaporator. The ethanolic extract (DIBE; 18.21 g) was suspended in 200 mL deionized water and partitioned sequentially with n-hexane, chloroform, and ethyl acetate using separating funnels in a stepwise manner. After vacuum filtration, the solutions were concentrated in a rotary vacuum evaporator. The n-hexane fraction (DIBH; 1.70 g), chloroform extract (DIBC; 2.44 g), ethyl acetate fraction (DIBEt; 8.55 g), and aqueous fraction (DIBW; 5.18 g) were dissolved in deionized water at 30 mg/mL concentration.

DIBEt was dissolved in deionized water and then diluted with HPLC-grade ethanol at 10 mg/mL concentration for PSI-tandem MS (MS/MS). The sample solution was vortexed for 1 min and sonicated for 5 min in a Powersonic 410 sonication bath (Hwashin Technology Co., Gyeonggi, Korea) for a homogeneous mixture.

### 2.2. PSI-MS

A 2 μL stock solution (10 mg/mL) was loaded using a disposable glass Pasteur pipette (Volac; Poulten & Graf Ltd., Barking, UK) onto the center of a chromatographic paper tip (Whatman 1 Chr., Kent, UK). The positive-ion mode of the Q-Exactive orbitrap MS (Thermo Fisher Scientific, Inc., Rockford, IL, USA) was used to collect the data over the range of *m/z* 50–600. To make a sharp tip, the chromatographic paper was cut into dimensions of 6 mm base and 14 mm height. A syringe pump (Fusion 100T; Chemyx, Stafford, TX, USA) was used to load the ethanol solvent onto the sample-loaded paper at a flow rate of 15 μL/min. A spray voltage of 4.5 kV was directly applied to the paper tip for the ionization of the sample. The other parameters for the PSI experiment were as follows: capillary temperature 300, S-lens RF level 50, mass resolution 140,000 (full-width at half-maximum), and maximum injection time 150 ms. The automatic gain control was set to 1 × 10^6^.

To perform the MS/MS experiments, three different stepped normalized collision energies (10, 30, and 50) were used with the same instrument. The instrument was operated in the positive-ion mode. The other operative parameters for the MS/MS experiments were as follows: sheath and auxiliary gas flow rate 10 and 0 (arbitrary units), respectively; spray voltage 3.60 kV; capillary temperature 300; and S-lens RF level 50.

### 2.3. Data Processing

Mass spectral data obtained from the orbitrap MS were processed using Xcalibur 3.1 with Foundation 3.1 (Thermo Fisher Scientific). Compounds were tentatively identified by matching their exact (calculated) masses of protonated (M + H) adducts with measured *m/z* values and PSI-MS/MS fragmentation patterns from the in-house MS/MS database, and online databases such as the Human Metabolome Database [15] and METLIN [16], and the literature. The compound structures were drawn using ChemDraw Professional 15.0 (PerkinElmer, Waltham, MA, USA).

### 2.4. Radical-Scavenging Activity Assays

DPPH, ABTS, superoxide, and hydroxyl radical-scavenging assays were conducted to evaluate the free radical-scavenging potential of DIB extract following the procedures outlined by Alam et al. [3]. Ascorbic acid and quercetin were used as positive controls for DPPH and ABTS and superoxide and hydroxyl radical-scavenging assays, respectively. The following equation was adapted to calculate the percent inhibition:

$$\text{Radical-scavenging}\;\mathrm{activity}\;\left(\%\;\mathrm{inhibition}\right)=\left(\frac{{\mathrm{Abs}}_{\mathrm{control}}-{\mathrm{Abs}}_{\mathrm{sample}}}{{\mathrm{Abs}}_{\mathrm{control}}}\right)\times 100$$

where Abs_control_ is the absorbance of the control sample and Abs_sample_ is the absorbance of the experimental sample. All samples were analyzed in triplicate.

To determine the reducing power potential, ferric reducing antioxidant power (FRAP) and cupric reducing antioxidant capacity (CUPRAC) assays were performed according to the method described by Alam et al. [17]. The reducing power potential was expressed as the ascorbic acid-equivalent antioxidant value (µM) calculated from the standard curve of ascorbic acid. The oxygen radical absorbance capacity (ORAC) assay [18] was performed using Trolox, a water-soluble analog of vitamin E, as a positive control. The antioxidant potentiality was calculated as the Trolox-equivalent antioxidant value (µM).

### 2.5. Cell Culture and Cell Viability Assay

RAW 264.7 cells (American Type Culture Collection, Rockville, MD, USA) were maintained in Dulbecco’s modified Eagle medium (DMEM) supplemented with 10% fetal bovine serum (FBS) and streptomycin-penicillin (100 µg/mL each; Hyclone, Logan, UT, USA) at 37 °C and 5% CO_2_. The cells were seeded in 96-well plates (5 × 10^5^ cells/mL) for 24 h and subsequently treated with DIBEt (1–100 µg/mL) for the next 24 h. Cell viability was measured using the 3-(4,5-dimethylthiazol-2-yl)-2,5-diphenyltetrazolium bromide (MTT) assay, as described previously [19].

### 2.6. Measurement of Intracellular ROS

The generation of *tert*-butyl hydroperoxide (t-BHP)-induced ROS as a cellular oxidative stress biomarker was determined by the 2′,7′-dichlorofluorescein diacetate (DCFH-DA) method [3].

### 2.7. Reverse Transcription-Polymerase Chain Reaction (RT-PCR)

Total RNA was isolated using TRIzol (Invitrogen, Carlsbad, CA, USA). RT & Go Mastermix (MP Biomedicals, Seoul, Korea) was used to prepare cDNA by implementing the manufacturer’s protocols. As described in Supplementary Table S1, various primers were used to perform RT-PCR using a PCR Thermal Cycler Dice TP600 (Takara Bio, Inc., Otsu, Japan) [17].

### 2.8. Western Blot Analysis

Cells were lysed and harvested using radioimmunoprecipitation assay buffer. Nuclear and cytosolic proteins were extracted by applying the nuclear and cytoplasmic extraction kit (Sigma-Aldrich, St. Louis, MO, USA). The bicinchoninic acid protein assay kit (Pierce, Rockford, IL, USA) was used to confirm the protein content. An adequate amount of protein (30 μg) was subjected to Western blot analysis, as described in a previous report using various antibodies (Supplementary Table S2) [20].

### 2.9. Statistical Analysis

Statistical analysis was performed using SigmaPlot version 12.5 (Systat Software, Inc., Chicago, IL, USA). Data were expressed as mean ± standard deviation (SD; *n* = 3). One-way analysis of variance (ANOVA) followed by Dunnett’s multiple comparison test was performed to determine the significance of the differentiation and fusion indices. *p* < 0.05 was considered significant.

## 3. Results and Discussion

### 3.1. Identification of Secondary Metabolites of DIBEt

**Figure 1:**
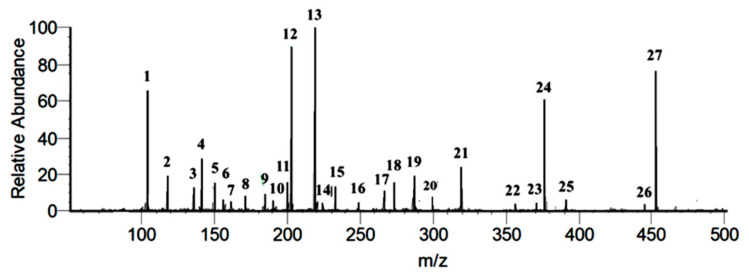
PSI-MS parent ion peak of the identified compounds of DIBEt.

The identification and characterization of the related compounds from DIBEt were performed in two steps. In the first step, PSI-MS was used to identify the major *m/z* peaks with a full-scan MS, and then characterized using PSI-MS/MS to obtain the MS/MS fragment of the obtained *m/z* from the first step. Figure 1 corresponds to the total ion chromatogram of DIBEt in PSI-MS in positive-ion mode, revealing 27 secondary metabolites presented with their molecular formula, monoisotopic mass of experimental ions, and calculated ions in positive modes (Table 1). All compounds identified in DIBEt were classified into nitrogen compounds, phenolic acids, flavonoids, amino acids, triterpenoids, and others.

**Figure 2:**
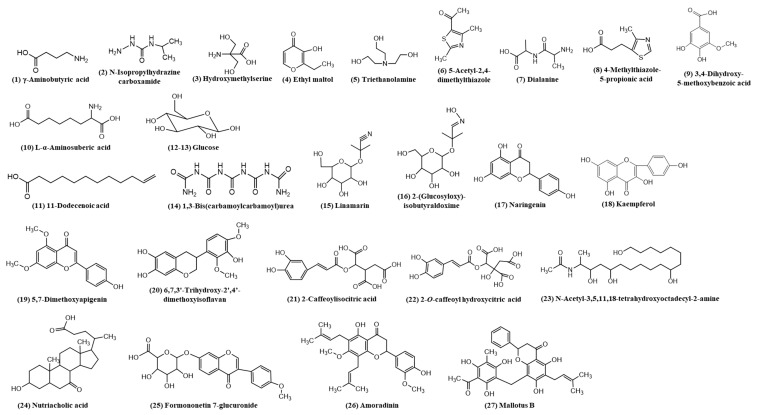
Chemical structures of the identified compounds of DIBEt by PSI-MS/MS.

Three phenolic acids (3,4-dihydroxy-5-methoxybenzoic acid, 2-caffeoylisocitric acid, and 2-*O*-caffeoylhydroxycitric acid) and seven flavonoids (naringenin, kaempferol, 5,7-dimethoxyapigenin, 6,7,3′-trihydroxy-2′,4′-dimethoxyisoflavan (bryaflavan), formononetin 7-glucoronide, amoradinin, and mallotus B (isoallorottlerin), along with the Na and K adducts of glucose) were identified. Twelve nitrogen compounds [γ-aminobutyric acid (GABA), *N*-isopropylhydrazinecarboxamide, hydroxymethylserine, triethanolamine, 5-acetyl-2,4-dimethylthiazole, dialanine, 4-methylthiazole-5-propionic acid, L-α-aminosuberic acid, 1,3-bis(carbamoylcarbamoyl)urea (carbonyldibiuret), linamarin, 2-(glucosyloxy)isobutyraldoxime, and *N*-acetyl-3,5,11,18-tetrahydroxyoctadecyl-2-amine] were also confirmed. One fatty acid (11-dodecenoic acid) and one triterpenoid (nutriacholic acid) were confirmed for the first time in this genus (Figure 2). Peaks 1–3, 5–8, 10, 14–16, and 23 were characterized as nitrogen compounds, such as GABA, *N*-isopropylhydrazinecarboxamide, hydroxymethylserine, triethanolamine, 5-acetyl-2,4-dimethylthiazole, dialanine, 4-methylthiazole-5-propionic acid, L-α-aminosuberic acid, 1,3-bis(carbamoylcarbamoyl)urea, linamarin, 2-(glucosyloxy)isobutyraldoxime, and *N*-acetyl-3,5,11,18-tetrahydroxyoctadecyl-2-amine, with the parent ion peak at *m/z* 104.1075, 118.0866, 136.0619, 150.1131, 156.0428, 161.0966, 172.0434, 190.1081, 233.0633, 248.1138, 266.1233, and 376.2597, respectively [21]. The detailed fragmentation patterns are given in Supplementary Figure S1. Seven flavonoids [naringenin (17), kaempferol (18), 5,7-dimethoxyapigenin (19), 6,7,3′-trihydroxy-2′,4′-dimethoxyisoflavan (20), formononetin 7-glucoronide (25), amoradinin (26), and mallotus B (27)] were also confirmed. Naringenin and kaempferol yielded a major fragment ion at *m/z* 153.01 and 119.05 and/or 121.02 due to ^1,3^A and ^0,2^B fragmentation, respectively. Polyphenolics also produced at *m/z* (M + H-44 u) and (M + H-18 u) by losing CO_2_ and water molecules, respectively, in positive-ion mode due to the abundance of carboxyl or hydroxyl groups. The detailed fragmentation patterns are given in Supplementary Figure S2. Furthermore, peaks 9, 21, and 22 were confirmed as 3,5-dihydroxy-4-methoxybenzoic acid, 2-caffeoylisocitric acid, and 2-*O*-caffeoylhydroxycitric acid with the parent ion peak at *m/z* 185.0445, 355.0697, and 371.0754, respectively (fragmentation patterns in Supplementary Figure S3). Peak 24 was suggested as nutriacholic acid [*m/z* 391.2841 (M + H)] and yielded a major fragmentation ion at *m/z* 207.14 due to the cleavage of C_8_–C_14_ and C_9_–C_11_ followed by water loss at *m/z* 189.13 (Supplementary Figure S4) [22].

### 3.2. Radical-Scavenging Activities of DIB Extracts

**Figure 3:**
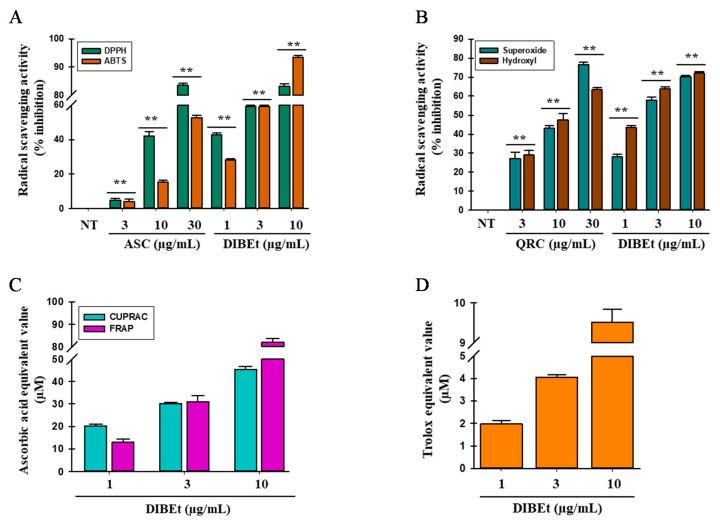
**Radical-scavenging effects of DIBEt.** DPPH and ABTS (**A**) and superoxide and hydroxyl (**B**) radical-scavenging activities of DIBEt. Ascorbic acid (ASC) and quercetin (QRC) were considered as standard antioxidant molecules. The reducing power of DIBEt was examined by CUPRAC, FRAP, and ORAC assays (**C**). The ascorbic acid-equivalent antioxidant capacity was calculated for CUPRAC and FRAP assays, and (**D**) the ORAC activity was expressed as the Trolox-equivalent antioxidant capacity. Mean ± SD (*n* = 3). ** *p* < 0.01.

Various molecular mechanisms and/or the synergism between them may cause the attribution of antioxidant activity of the secondary metabolites present in plants. Thus, the evaluation of the antioxidant activity of plant extracts should be performed via several methods. DPPH, ABTS, superoxide and hydroxyl radical-scavenging assays, and FRAP, CUPRAC, and ORAC assays were performed to assess the antioxidant potential of various organic and aqueous DIB extracts. All organic and aqueous DIB extracts significantly scavenged DPPH and ABTS radicals in a dose-dependent manner (Figure 3A; Supplementary Figure S5). DIBEt showed 7.34- and 3.81-fold higher DPPH and ABTS radical-scavenging activities, respectively, than ascorbic acid used as a positive control, with IC_50_ of 1.87 ± 0.13 and 7.58 ± 0.10 µg/mL for DIBEt and 13.79 ± 0.87 and 28.90 ± 0.16 µg/mL for ascorbic acid, respectively. Other extracts showed DPPH and ABTS radical-scavenging activities in the following order: DIBE (IC_50_, 5.12 ± 0.68 µg/mL) > DIBW (IC_50_, 5.82 ± 0.18 µg/mL) > DIBC (IC_50_, 50.13 ± 1.08 µg/mL) > DIBH (IC_50_, 63.79 ± 2.19 µg/mL) and DIBE (IC_50_, 9.95 ± 0.05 µg/mL) > DIBW (IC_50_, 17.54 ± 1.15 µg/mL) > DIBC (IC_50_, 91.00 ± 0.86 µg/mL) > DIBH (IC_50_, >100 µg/mL), respectively. Previous studies revealed that the methanolic extracts of *D. indica* fruits showed strong DPPH radical-scavenging activity followed by petroleum ether, ethyl acetate, and water extract with IC_50_ of 31.25, 65.77, 97.25, and 106.95 µg/mL, respectively [12,23]. Compared to previous studies, this study revealed that DIB is more powerful to scavenge DPPH radicals. The superoxide and hydroxyl radical-scavenging abilities of DIB extracts were evaluated by the PMS-NADH superoxide-generating system and Fenton reaction in a dose-dependent manner, respectively (Figure 3B; Supplementary Figure S6). DIBEt had 5.70- and 7.10-fold higher superoxide and hydroxyl radical-scavenging potential than quercetin, with IC_50_ of 2.47 ± 0.05 and 1.58 ± 0.06 µg/mL for DIBEt and 14.12 ± 0.77 and 11.21 ± 1.06 µg/mL for quercetin, respectively. Other extracts had superoxide and hydroxyl radical-scavenging activities in the following order: DIBE (IC_50_, 14.78 ± 1.15 µg/mL) > DIBW (IC_50_, 14.48 ± 0.17 µg/mL) > DIBC (IC_50_, >100 µg/mL) > DIBH (IC_50_, >100 µg/mL) and DIBE (IC_50_, 7.85 ± 0.02 µg/mL) > DIBW (IC_50_, 9.54 ± 0.09 µg/mL) > DIBC (IC_50_, 22.41 ± 2.17 µg/mL) > DIBH (IC_50_, 23.85 ± 0.47 µg/mL), respectively. Das et al. [23] reported that the methanolic extract of *D. indica* fruits had powerful superoxide and hydroxyl radical-scavenging activities with IC_50_ of 51.49 and 51.82 µg/mL, respectively. In contrast, this study showed that various organic and aqueous DIB extracts are more powerful to scavenge superoxide and hydroxyl radicals.

CUPRAC, FRAP, and ORAC assays were performed to determine whether DIBEt is capable to donate electrons and establish that DIBEt has a strong reducing power potential in a dose-dependent manner (Figure 3C; Supplementary Figure S7). At 10 µg/mL, DIBEt showed 34.52 ± 0.37 and 81.37 ± 0.57 µM ascorbic acid-equivalent reducing power for CUPRAC and FRAP assays, respectively. Other extracts showed ascorbic acid-equivalent reducing power activities in the following order: DIBE (30.64 ± 0.58 µM) > DIBW (24.51 ± 1.13 µM) > DIBC (11.85 ± 2.03 µM) > DIBH (4.22 ± 0.28 µM) and DIBE (21.08 ± 1.25 µM) > DIBW (19.72 ± 0.80 µM) > DIBC (6.80 ± 0.89 µM) > DIBH (3.30 ± 1.57 µM) for CUPRAC and FRAP assays, respectively. DIBEt showed 9.43 ± 1.97 µM Trolox-equivalent antioxidant capacity at 10 µg/mL in the ORAC assay. Other extracts showed Trolox-equivalent antioxidant capacity in the following order: DIBE (7.87 ± 0.04 µM) > DIBW (5.55 ± 0.27 µM) > DIBC (3.10 ± 0.20 µM) > DIBH (2.74 ± 0.13 µM). Based on these interpretations, DIBEt has a very strong potential to donate/transfer hydrogen/electrons to oxidants to neutralize them.

Studies revealed that phenolic compounds, such as phenol and flavonoids, have strong redox properties capable of quenching singlet and triplet oxygen, adsorbing and neutralizing free radicals, and/or decomposing peroxides, resulting in superior antioxidant potential [17,24,25]. Thus, total phenolic and flavonoid contents were found in the plant chosen for the study (Supplementary data S8). In addition, Pearson coefficient (*ρ*) and linear regression analyses were performed to correlate the polyphenol, flavonoid, and antioxidant activities of DIB extracts. The results showed substantial correlation for DPPH, ABTS, and hydroxyl radical-scavenging activities (*ρ* = −0.939, −0.918, and −0.853, respectively) and moderate correlation for superoxide radical-scavenging activity (*ρ* = −0.622). A negative *ρ*-value (−1) stands for a perfect positive correlation, as a correlation between polyphenol and free radical-scavenging abilities was found using IC_50_. The data were also supported by previous studies describing that the antioxidant activity is highly influenced by the presence of total phenol content, and has a linear correlation between phenolic content and antioxidant activity, but the total flavonoid content provided a mixed function [3,26].

The radical-scavenging and reducing power activities of the identified constituents of DIBEt were also tested. The identified compounds showed a strong radical-scavenging activity (IC_50_) in the order of naringenin > kaempferol > 3,4-dihydroxy-5-methoxybenzoic acid > ethyl maltol > dialanine > GABA ≅ linamarin. Naringenin also showed the highest reducing power activity, with an ascorbic acid-equivalent of 33.32 ± 0.31 and 54.34 ± 0.29 µM for CUPRAC and FRAP assays, respectively, followed by kaempferol > 3,4-dihydroxy-5-methoxybenzoic acid > ethyl maltol > dialanine > GABA ≅ linamarin (Table 2).

Studies revealed that 3,4-dihydroxy-5-methoxybenzoic acid and 2-*O*-caffeoylhydroxycitric acid have strong free radical-scavenging activities, with IC_50_ of 10.69 and 6.05 µg/mL and 10.23 and 4.32 µg/mL for DPPH and ABTS assays, respectively [27,28]. Flavonoids, such as naringenin, kaempferol, and 6,7,3′-trihydroxy-2′,4′-dimethoxyisoflavan, also have very strong free radical-scavenging activities as reported by previous studies [29,30]. Triethanolamine, dialanine, and linamarin have also been studied for their antioxidant properties [31,32]. GABA- and mallotus B-containing plant extracts also showed excellent antioxidant properties and protect RIN-m5F pancreatic cells from hydrogen peroxide-induced oxidative stress [33,34].

### 3.3. DIBEt Attenuates t-BHP-Induced Cellular Oxidative Stress

**Figure 4:**
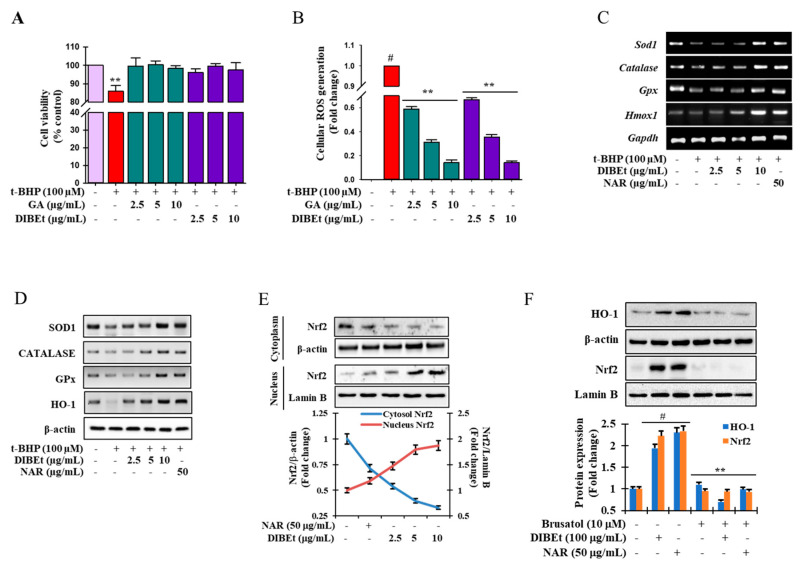
Protective effects of DIBEt against t-BHP-induced cell toxicity and intracellular ROS generation through the up-regulation of antioxidant enzymes via Nrf2 activation. Cells were treated with DIBEt and gallic acid (GA) at the indicated concentrations for 12 h and then exposed to 100 μM t-BHP for 6 h. Cell viability percentage (**A**) and intracellular ROS (**B**) were determined by MTT assay and the DCFH-DA method, respectively. Mean ± SD (*n* = 3). # *p* < 0.001, compared to no treatment; ** *p* < 0.05, compared to t-BHP treatment. SOD1, catalase, GPx, and phase II antioxidant enzyme (such as HO-1) mRNA (**C**) and protein expression (**D**) were analyzed by RT-PCR and Western blot, respectively. Nrf2 protein expression (**E**) was measured by Western blot. Cells were treated with an Nrf2 inhibitor (brusatol) with and without DIBEt and naringenin (NAR). Nrf2 and HO-1 protein levels were analyzed by Western blot (**F**). Mean ± SD (*n* = 3). # *p* < 0.001, compared to no treatment; ** *p* < 0.05, compared to sample treatment. (+): presence; (-): absence.

Among all extracts, DIBEt has the highest antioxidant potential triggered to evaluate the potential of DIBEt to scavenge oxidative stress-induced cellular ROS generation with cellular toxicity. t-BHP, a short-chain analog of lipid peroxide, is widely accepted as a model substance to induce oxidative stress in cells and tissues and evaluate the molecular mechanisms of cellular alterations caused by oxidative stress [35]. More than 95% of cell viability was noted at DIBEt concentrations up to 50 µg/mL (Figure 4A; Supplementary Figure S9). In addition, Figure 4B demonstrates that DIBEt has an immense potential to attenuate oxidative stress-induced cellular ROS generation in a concentration-dependent manner with that of gallic acid (30 μg/mL) without showing cellular toxicity. Excessive ROS formation triggers oxidative stress, leading to cell death. Detailed research has revealed that antioxidants mitigated the deleterious effects of ROS and retard many effects that cause cellular death [3,36,37].

### 3.4. Effects of DIBEt on Antioxidant Enzyme Expression in RAW 264.7 Cells

The first-line antioxidant defense system, including superoxide dismutase, catalase, glutathione peroxidase (GPx), and glutathione, critically maintains cellular redox homeostasis [37]. Various stimuli can induce HO-1 expression, conferring cell protection from oxidative stress by maintaining antioxidant/oxidant homeostasis [38]. In Figure 4C,D, the mRNA expression and protein levels of the first-line antioxidant enzymes SOD1, catalase, and GPx-1 and the phase II enzyme HO-1 were extremely extenuated in t-BHP-treated cells, respectively, but DIBEt treatment dose-dependently reversed this trend. Naringenin also enhanced the mRNA expression and protein levels of first-line antioxidant enzymes in the t-BHP model. These data suggest that increased mRNA and protein levels of antioxidant enzymes by DIBEt plays a critical role in cellular homeostasis and protects against oxidative stress-induced cell death.

Studies revealed that medicinal plants or foods possess abundant polyphenolic compounds that boost SOD1, CAT, and GPx activities, decreasing oxidative stress [39,40]. Evidence showed that polyphenolic acids, such as naringenin, kaempferol, apigenin, and formononetin aglycone, and their glycosidic form and caffeoylisocitric acid have a potential to boost first-line antioxidant proteins, leading to cell protection against oxidative stress [41,42,43,44,45]. Therefore, the presence of phenolic acids and flavonoids in DIBEt may play a major role in boosting the expression of first-line antioxidant enzymes/proteins as the principal mechanism accounting for protecting DIBEt against oxidative stress.

### 3.5. Effects of DIBEt on Phase II Enzymes Mediated by Nrf2 Nuclear Translocation in RAW 264.7 Cells

An important transcription factor, Nrf2, binds to the ARE in the promoter regions of cytoprotective genes and acts as a master regulator of antioxidative responses [46]. Thus, immunoblotting was performed to evaluate the role of DIBEt on Nrf2 regulation. In Figure 4E, the nuclear Nrf2 content was markedly increased in association with decreased cyto-Nrf2 levels after DIBEt treatment. Furthermore, brusatol, a pharmacological inhibitor of Nrf2, was used to confirm the role of DIBEt on the activation of phase II detoxifying enzymes through Nrf2 regulation. Nrf2 protein levels were greatly diminished by brusatol treatment, which was not reestablished despite the application of DIBEt and naringenin (Figure 4F). Furthermore, DIBEt and naringenin treatment was also unable to restore the basal HO-1 protein levels in brusatol-treated cells (Figure 4F). This observation conferred that DIBEt might disrupt the proteasomal degradation of Nrf2 in the cytoplasm by Keap1 and facilitate Nrf2 nuclear translocation, resulting in the up-regulation of HO-1 expression. Studies revealed that extracts from various medicinal plants or foods, such as *Nymphaea nouchali* flowers, *Lannea coromandelica* bark, and *Ginkgo biloba* bark, can activate Nrf2-mediated phase II enzyme expression in RAW 264.7, Hepa-1c1c7, and Hep G2 cells [3,17,47]. Furthermore, naringenin, kaempferol, apigenin, and formononetin aglycone and their glycosidic form can modulate the Nrf2/ARE/HO-1 signaling cascade, leading to the attenuation of oxidative stress-mediated melanocytes and kidney and neuronal death [43,44,45].

### 3.6. DIBEt Regulates Nrf2 Translocation Via Activation of MAPK to Lessen Oxidative Stress

**Figure 5:**
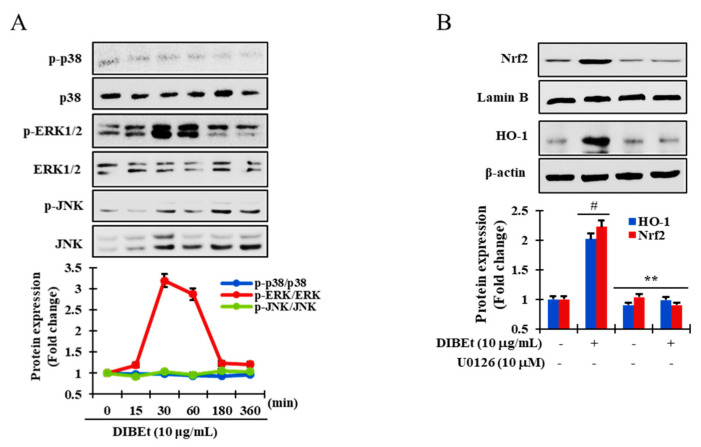
**DIBEt facilitates Nrf2 translocation by activating ERK1/2.** RAW 264.7 cells were pretreated with DIBEt (10 µg/mL) at the indicated times. Immunoblotting was performed to evaluate kinase activity (**A**). Cells were treated with DIBEt in the presence and absence of the specific inhibitor U0126. The protein levels of Nrf2 and HO-1 were analyzed by Western blot (**B**). Mean ± SD (*n* = 3). # *p* < 0.001; ** *p* < 0.05, compared to no treatment. Statistical analysis was performed using one-way ANOVA.

MAPKs act as a downstream effector in antioxidant responses. The activation of MAPKs can manifest the activation of Nrf2. In-vitro and in-vivo studies have revealed that extracellular signal-regulated kinase (ERK), JNK, and p38 MAPK positively regulate ARE-containing reporter or detoxifying genes via Nrf2-dependent mechanisms [48,49]. In Figure 5A, ERK1/2 was phosphorylated from 15 to 180 min and peaked at 30 min after DIBEt exposure, whereas p38 and JNK phosphorylation was absent in DIBEt-treated cells. Furthermore, to confirm the role of ERK1/2 phosphorylation in Nrf2 translocation to the nucleus and induction of HO-1 expression, U0126, a specific inhibitor of ERK1/2, was used in DIBEt-treated cells. As expected, in Figure 5B, Nrf2 nuclear translocation and subsequently HO-1 expression were successfully enhanced in DIBEt-treated cells, which were strongly mitigated in U0126-treated cells, suggesting that ERK activation plays a critical role in DIBEt-induced Nrf2 translocation into the nucleus and subsequent boost of HO-1 expression in RAW 264.7 cells. Moreover, according to previous reports, dietary antioxidants can cause MAPK activation accountable for cell protection against oxidative stress [50].

## 4. Conclusions

Oxidative stress is a major causative condition for the development and progression of numerous acute and chronic clinical disorders. Thus, antioxidants may cause health benefits as prophylactic agents. In this study, DIBEt contained various polyphenolic compounds as confirmed by PSI-MS/MS and showed superior antioxidant activity in cell-free and cellular levels. DIBEt treatment successfully lessened oxidative stress and cell death, most likely by (i) reducing ROS generation and (ii) boosting the expression of endogenous antioxidant enzymes and/or Nrf2-mediated HO-1 expression. DIBEt pretreatment may cause the activation of ERK1/2 signaling pathways involved in the cytoprotective effects of DIBEt. The findings provide new insights into the cytoprotective effects and mechanisms of DIB against oxidative stress, which may be used as treatment for oxidative stress-induced disorders.

## Figures and Tables

**Table 1:** Characterization of the secondary metabolites of DIBEt by PSI-MS/MS.

No.	Compound Name	MW	MM	EF	(M + H) *m/z* ^a^	(M + H) *m/z* ^b^	PSI-MS/MS
							(Positive Ionization)
1	γ-Aminobutyric acid (GABA)	103.121	103.063	C_4_H_9_NO_2_	104.108	104.071	87.04, 60.02, 58.07
2	N-Isopropylhydrazinecarboxamide	117.152	117.09	C_4_H_11_N_3_O	118.087	118.098	101.06, 59.07, 58.06
3	Hydroxymethylserine	135.119	135.053	C_4_H_9_NO_4_	136.062	136.061	119.035
4	Ethyl maltol	140.138	140.047	C_7_H_8_O_3_	141.055	141.055	140.06, 126.03, 123.04, 113.06, 108.02, 81.04
5	Triethanolamine	149.188	149.105	C_6_H_15_NO_3_	150.113	150.113	132.10, 120.10, 103.06, 88.07
6	5-Acetyl-2,4-dimethylthiazole	155.22	155.04	C_7_H_10_NOS	156.043	155.048	140.03, 122.07, 81.04
7	Dialanine	160.171	160.085	C_6_H_12_N_2_O_3_	161.097	161.092	145.06, 144.07, 131.06, 118.05, 101.07, 88.04, 72.04
8	4-Methylthiazole-5-propionic acid	171.214	171.035	C_7_H_9_NO_2_S	172.043	172.043	156.06, 141.08, 128.07, 113.07
9	3,5-Dihydroxy-4-methoxybenzoic acid	184.157	184.037	C_8_H_8_O_5_	185.045	185.045	170.02, 155.03, 126.02, 109.03, 95.05, 91.02, 77.04
10	L-α-Aminosuberic acid	189.211	189.1	C_8_H_15_NO_4_	190.108	190.108	173.08, 130.09, 128.07, 113.06, 101.06, 70.01
11	11-Dodecenoic acid	198.306	198.162	C_12_H_22_O_2_	199.169	199.169	181.16, 127.08, 95.09, 85.03
12	Glucose Na adduct				203.053	203.052	
13	Glucose K adduct				219.027	219.026	
14	1,3-Bis(carbamoylcarbamoyl)urea (Carbonyldibiuret)	232.156	232.056	C_5_H_8_N_6_O_5_	233.063	233.063	216.08, 188.09, 145.03, 119.01, 102.05
15	Linamarin	247.247	247.106	C_10_H_17_NO_6_	248.114	248.113	230.10, 182.08, 128.07, 115.04, 98.04
16	2-(Glucosyloxy) isobutyraldoxime	265.262	265.112	C_10_H_19_NO_7_	266.123	266.123	248.11, 230.10, 182.08, 128.07, 115.04, 98.04
17	Naringenin	272.256	272.068	C_15_H_12_O_5_	273.076	273.076	153.01, 147.04, 119.05
18	Kaempferol	286.239	286.048	C_15_H_10_O_6_	287.056	287.055	269.04, 141.05, 213.05, 165.02, 153.02, 137.02, 121.02
19	5,7-Dimethoxyapigenin	298.29	298.084	C_17_H_14_O_5_	299.056	299.091	271.10, 253.09, 179.03, 137.06, 123.04
20	6,7,3′-Trihydroxy-2′,4′-dimethoxyisoflavan (Bryaflavan)	318.325	318.11	C_17_H_18_O_6_	319.116	319.11	301.11, 245.08, 195.10, 167.07, 153.05, 149.05, 137.02
21	2-Caffeoylisocitric acid	354.267	354.059	C_15_H_14_O_10_	355.07	355.066	310.07, 121.03, 203.02, 192.03, 177.04
22	2-*O*-caffeoylhydroxycitric acid	370.26	370.053	C_15_H_14_O_11_	371.075	371.061	311.04, 279.05, 267.05, 237.04
23	N-Acetyl-3,5,11,18-tetrahydroxyoctadecyl-2-amine	375.52	375.295	C_20_H_41_NO_5_	376.26	376.303	358.30, 340.28, 226.18, 161.15, 147.14, 137.06, 123.04, 109.10
24	Nutriacholic acid	390.62	390.277	C_24_H_38_O_4_	391.284	391.284	361.27, 207.14, 189.13, 161.13, 149.13
25	Formononetin 7-glucoronide	444.38	444.105	C_22_H_20_O_10_	445.12	445.113	413.12, 251.07, 137.02, 123.04
26	Amoradinin	452.521	452.219	C_27_H_32_O_6_	453.231	453.231	391.15, 373.14, 361.14, 207.07, 191.07
27	Mallotus B (Isoallorottlerin)	518.554	518.194	C_30_H_30_O_8_	519.205	519.202	339.17, 237.11

^a^ Observed parent ion *m/z* (M + H); ^b^ Calculated parent ion *m/z* (M + H). MW, average molecular weight (g/mol); MM, monoisotopic molecular mass (g/mol); EF, elemental formula.

**Table 2:** Antioxidant activities of commercially available identified compounds from DIBEt.

	Compound Name	DPPH ^a^	ABTS ^a^	CUPRAC ^b^	FRAP ^b^
1	GABA	>100	>100	0.12 ± 1.51	0.91 ± 1.25
2	Ethyl maltol	29.42 ± 0.85	34.52 ± 0.35	21.05 ± 1.15	28.52 ± 0.55
3	Dialanine	85.69 ± 0.25	92.57 ± 0.53	09.55 ± 0.25	11.02 ± 1.52
4	3,4-Dihydroxy-5-methoxybenzoic acid	18.25 ± 1.52	12.05 ± 0.45	25.15 ± 0.85	35.25 ± 0.89
5	Linamarin	>100	>100	0.32 ± 0.94	0.71 ± 0.65
6	Naringenin	4.52 ± 0.12	3.98 ± 0.78	33.32 ± 0.31	54.34 ± 0.29
7	Kaempferol	10.32 ± 0.54	5.21 ± 0.45	31.02 ± 0.57	45.53 ± 0.92

^a^ Radical-scavenging activities as IC_50_ (µg/mL). ^b^ Ascorbic acid-equivalent reducing power (µM). GABA, ethyl maltol, dialanine, 3,4-dihydroxy-5-methoxybenzoic acid, naringenin, and kaempferol were purchased from Sigma-Aldrich (catalog nos. A2129, W348708, A9502, CDS003720, N5893, and 60010, respectively). Linamarin was purchased from Cayman Chemical (Ann Arbor, MI, USA). All standards of purity were >98%.

## Supplementary Materials

The following are available online at https://www.mdpi.com/2076-3921/9/11/1099/s1, Figure S1: Mass fragmentation of nitrogen compounds in DIB extracts. Figure S2: Mass fragmentation of flavonoids in DIB extracts. Figure S3: Mass fragmentation of phenolic acids in DIB extracts. Figure S4: Mass fragmentation of ethyl maltol, 11-dodecenoic acid, and the triterpenoid nutriacholic acid in DIB extracts. Figure S5: DPPH and ABTS radical-scavenging activities of various organic and aqueous DIB extracts. Figure S6: Superoxide and hydroxyl radical-scavenging activities of various organic and aqueous DIB extracts. Figure S7: CUPRAC and FRAP activities of various organic and aqueous DIB extracts. Figure S8: Total phenol (A) and flavonoid (B) content of various organic and aqueous DIB extracts. Figure S9: Cell viability of DIBEt. Table S1: List of primer sequences used in this study. Table S2: List of antibodies used in this study.
