# Supplementary material for: Phytochemical Characterization of Dillenia indica L. Bark by Paper Spray Ionization-Mass Spectrometry and Evaluation of Its Antioxidant Potential Against t-BHP-Induced Oxidative Stress in RAW 264.7 Cells
Source: Antioxidants (Basel). 2020 Nov 9;9(11):1099. doi: 10.3390/antiox9111099 (PMC7695284; doi:10.3390/antiox9111099)
Supplement: Supplementary file 1 [file antioxidants-09-01099-s001.pdf]

Supplementary Data

Phytochemical characterization of *Dillenia indica* L. bark by paper spray ionization mass spectrometry and evaluation of its antioxidant potential against t-BHP-induces oxidative stress in RAW264.7 cells

Md Badrul Alam[a,b,1], Arif Ahmed[c,1], Syful Islam[c], Hee-Jeong Choi[a], Md Abdul Motin[d], Sunghwan Kim[c,e,*], Sang Han Lee[a,b,f]**

[a]Department of Food Science and Biotechnology, Graduate School, Kyungpook National University, Daegu 41566, Korea

[b]Food and Bio-Industry Research Institute, Inner Beauty/Antiaging Center, Kyungpook National University, Daegu 41566, Korea

[c]Department of Chemistry, Kyungpook National University, Daegu, 41566, Republic of Korea.

[d]Department of Chemistry, University of California, Riverside, CA 9252, USA.

[e]Mass Spectrometry Converging Research Center and Green-Nano Materials Research Center, Daegu, 41566, Republic of Korea.

[f]knu BnC, Daegu 41566, Korea

[1]These authors have equal contribution

[*]**Corresponding authors:**

[**] **Correspondence to:** **Dr. Sang Han Lee;** Department of Food Science and Biotechnology, Kyungpook National University, Daegu 41566, Korea, Phone: (82)053-950-7754 (Office); (82)010-2537-7659 (Mobile); Fax: 053-950-6772; Email: sang@knu.ac.kr; **Dr. Sunghwan Kim**, Department of Chemistry, Kyungpook National University, Daegu, 41566, Republic of Korea, Phone: (82)053-950-7754 (Office); (82)010-2537-7659 (Mobile); Fax: 053-950-6772; Email: sunghwank@knu.ac.kr

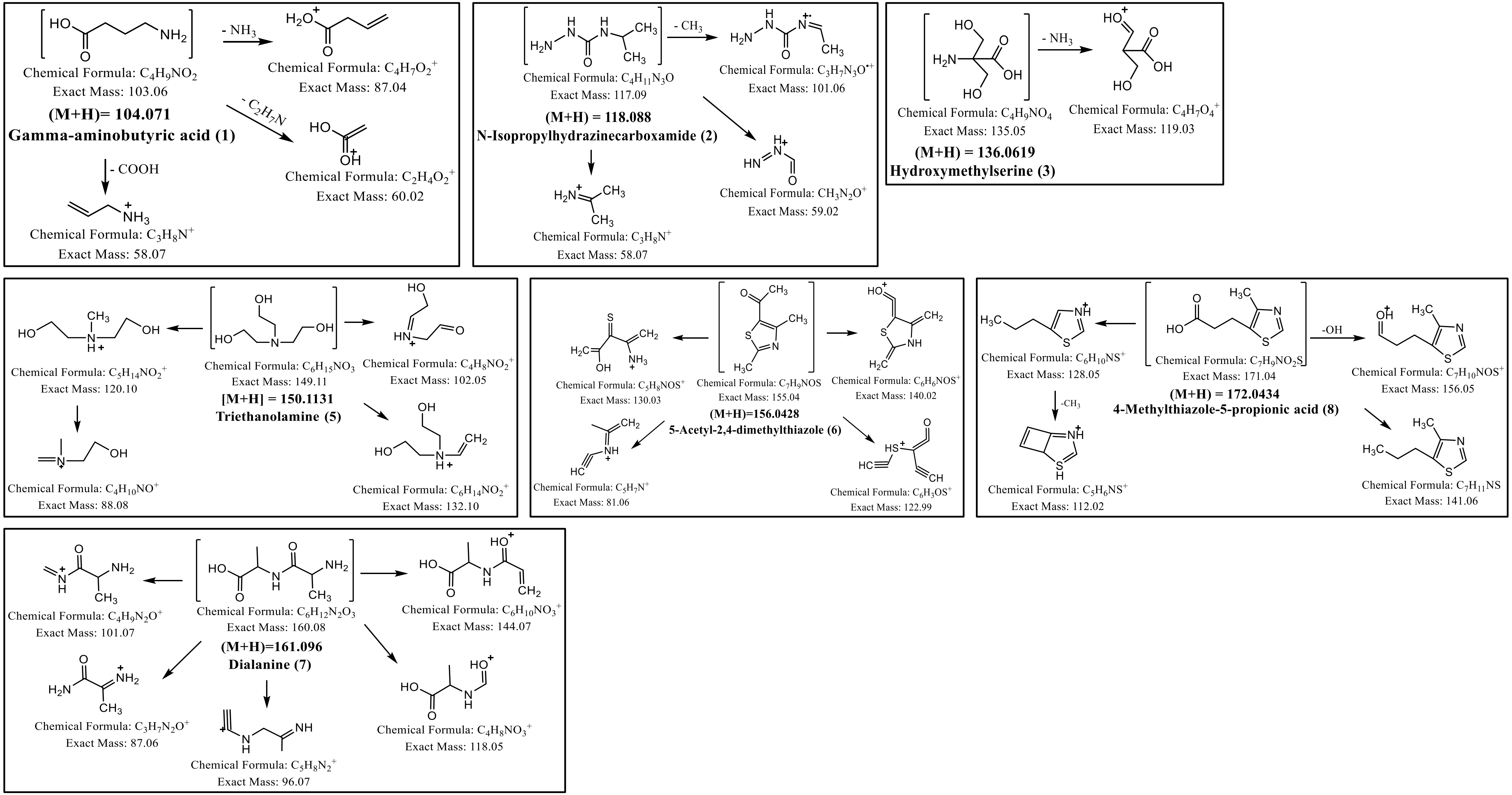

Figure S1: Mass fragmentation of Nitrogen compounds in *D. indica* bark extracts. (continued)

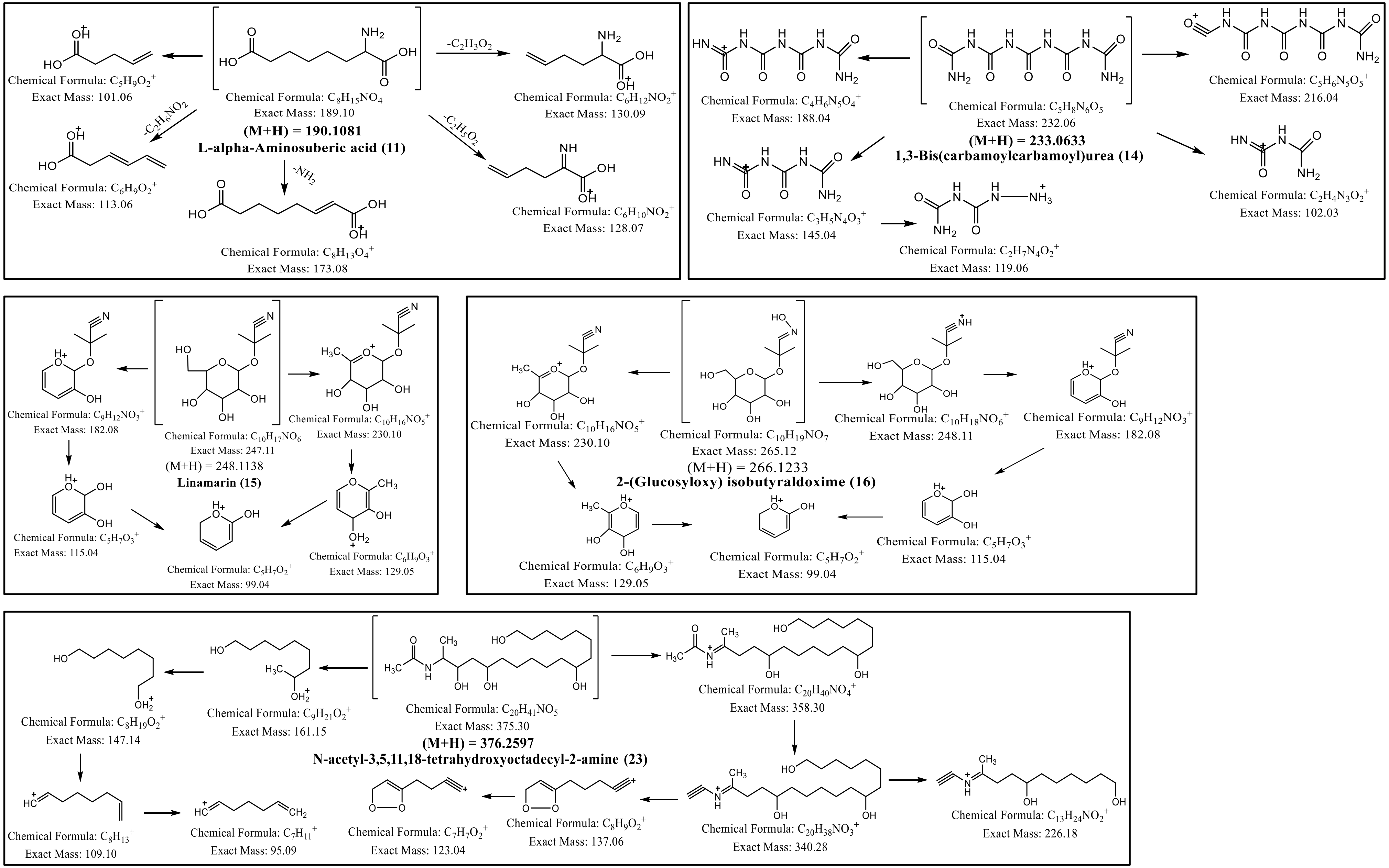

Figure S1: Mass fragmentation of Nitrogen compounds in *D. indica* bark extracts.

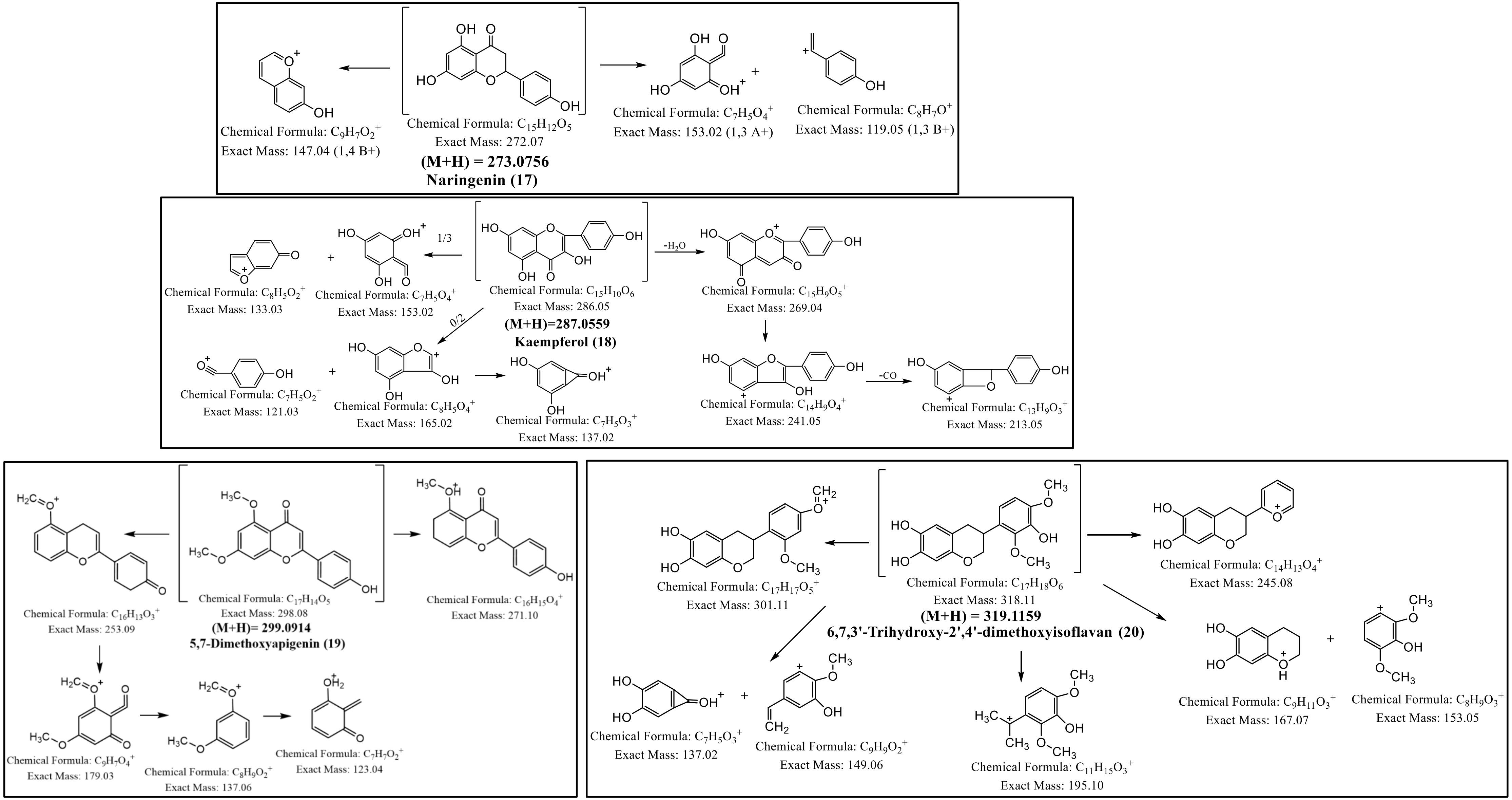

Figure S2: Mass fragmentation of flavonoids in *D. indica* bark extracts (continued).

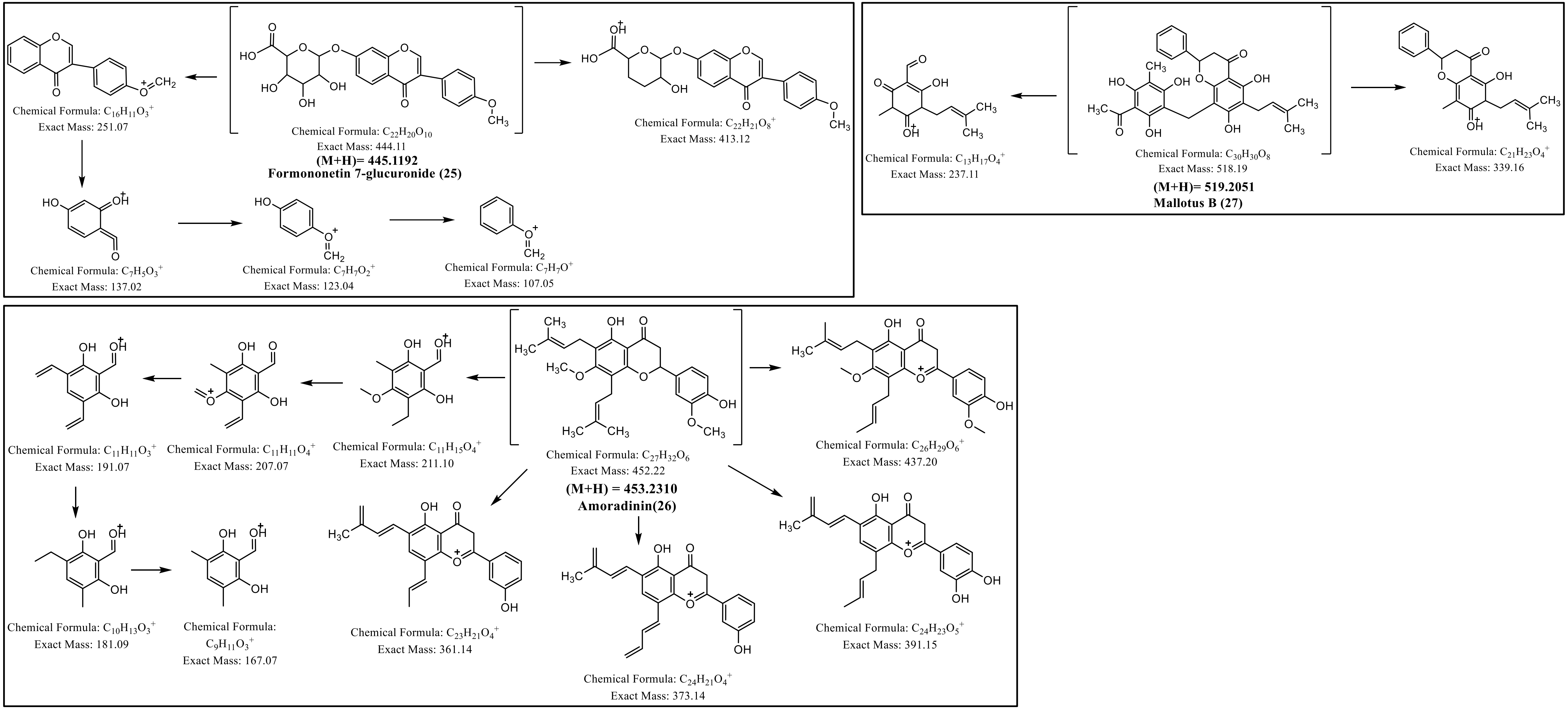

Figure S2: Mass fragmentation of flavonoids in *D. indica* bark extracts.

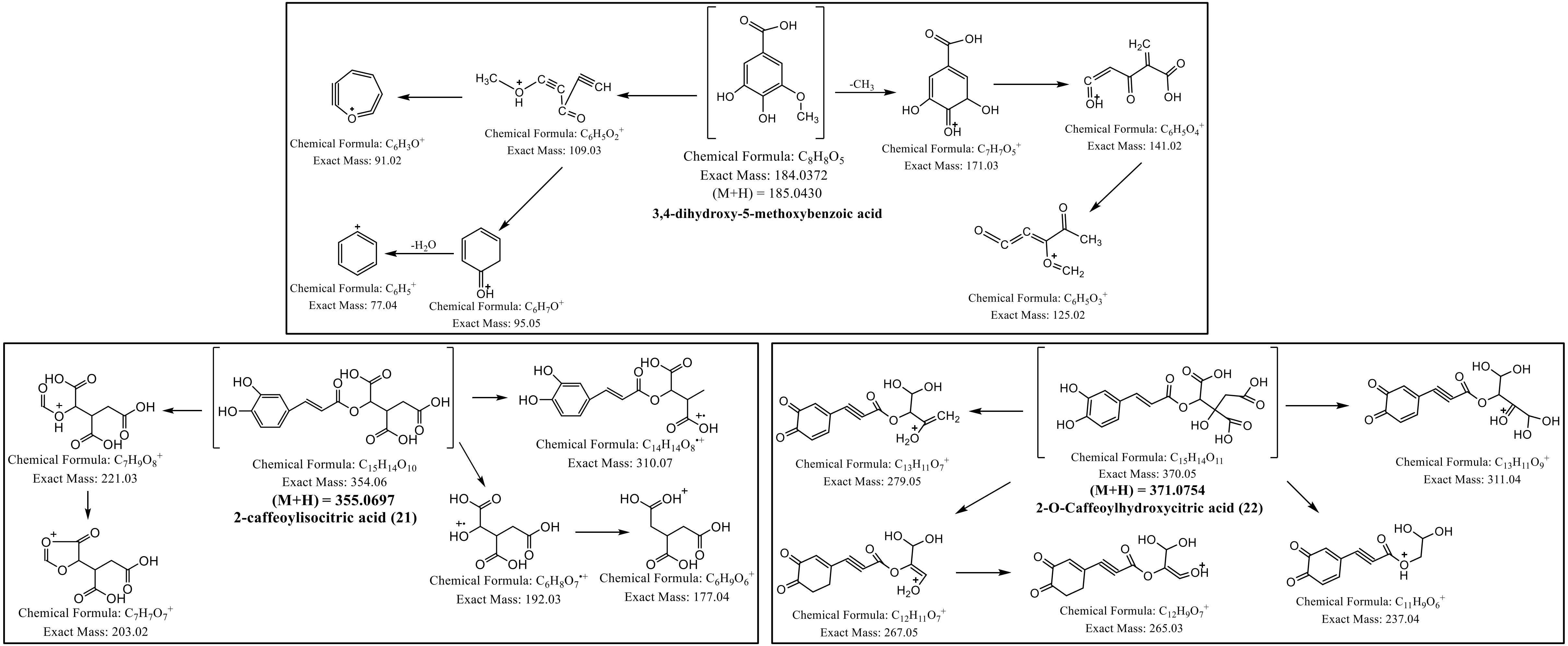

Figure S3: Mass fragmentation of phenolic acids in *D. indica* bark extracts.

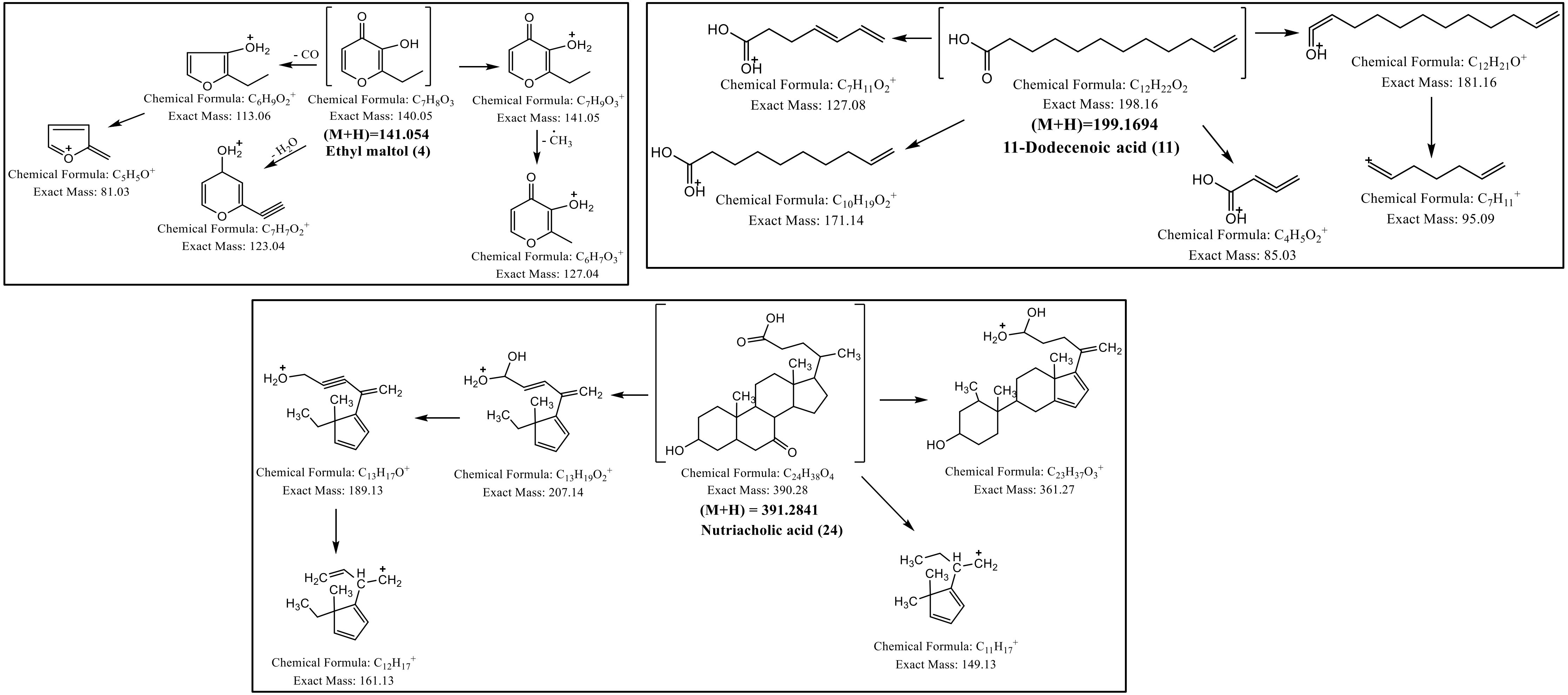

Figure S4: Mass fragmentation of ethyl maltol, 11-dodecenoic acid and triterpenoid nutriacholic acid in *D. indica* bark extracts.

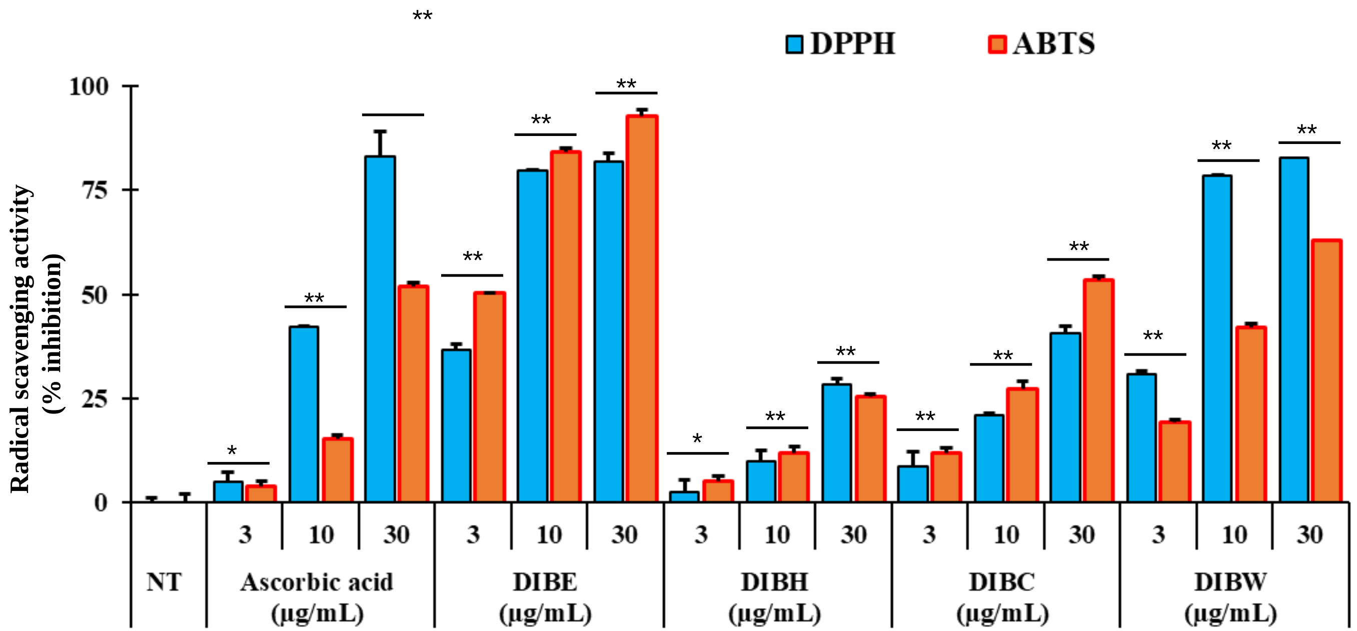

Figure S5: DPPH- and ABTS-radical scavenging activities of various organic and aqueous extracts of *D. indica* bark. DIBE: ethanol extract; DIBH: hexane extract; DIBC: chloroform extract; and DIBW: aqueous extract of *D. indica* bark. Values are expressed as the mean ± SD (n = 3). *p < 0.05 **p < 0.01.

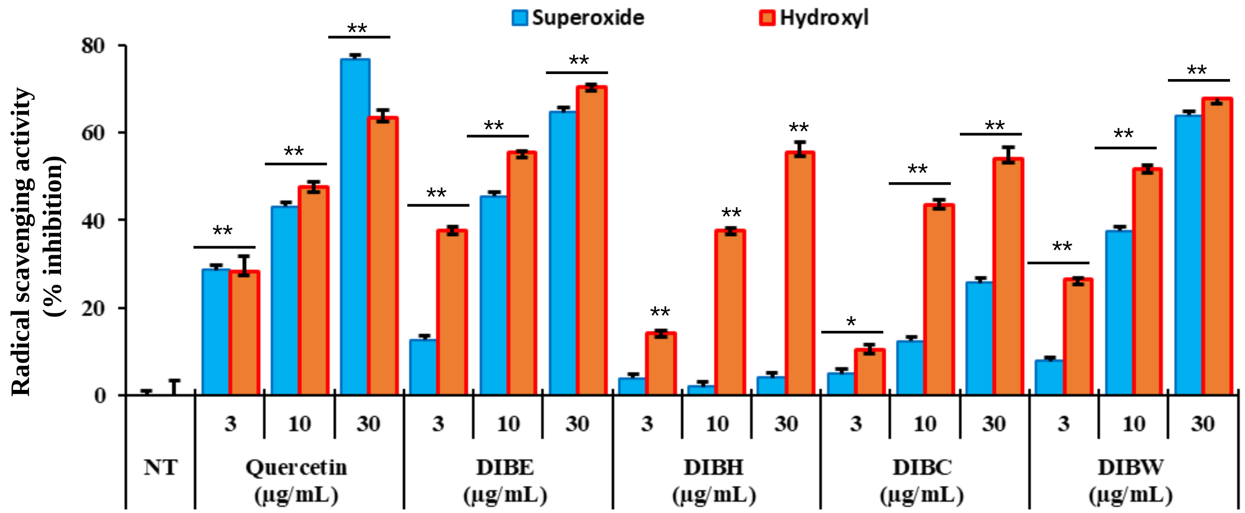

Figure S6: Superoxide- and hydroxyl-radical scavenging activities of various organic and aqueous extracts of *D. indica* bark. DIBE: ethanol extract; DIBH: hexane extract; DIBC: chloroform extract; and DIBW: aqueous extract of *D. indica* bark. Values are expressed as the mean ± SD (n = 3). *$p < 0.05$ **$p < 0.01$

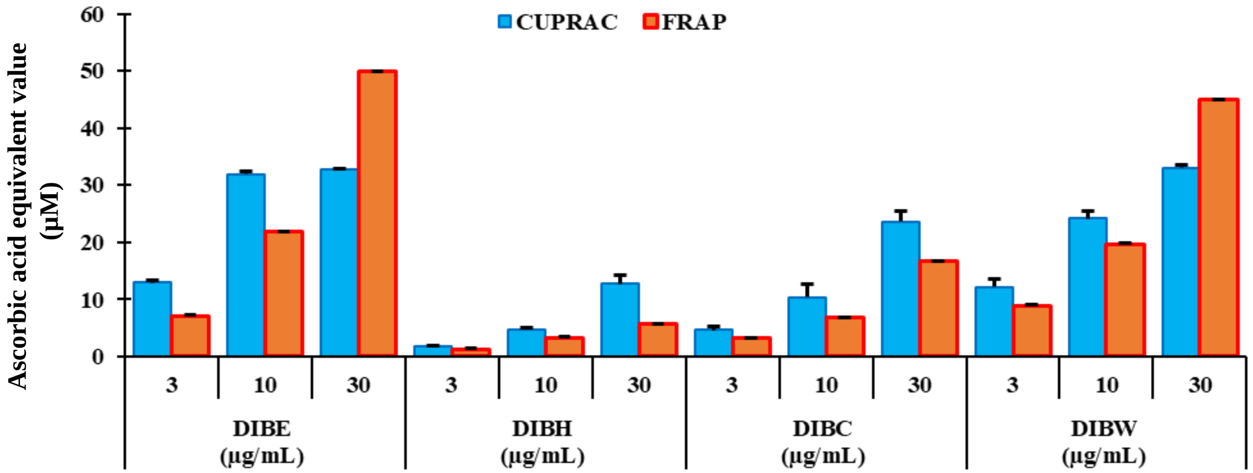

Figure S7: CUPRAC and FRAP activities of various organic and aqueous extracts of *D. indica* bark.
DIBE: ethanol extract; DIBH: hexane extract; DIBC: chloroform extract; and DIBW: aqueous extract of *D. indica* bark.
Values are expressed as the mean ± SD (n = 3).

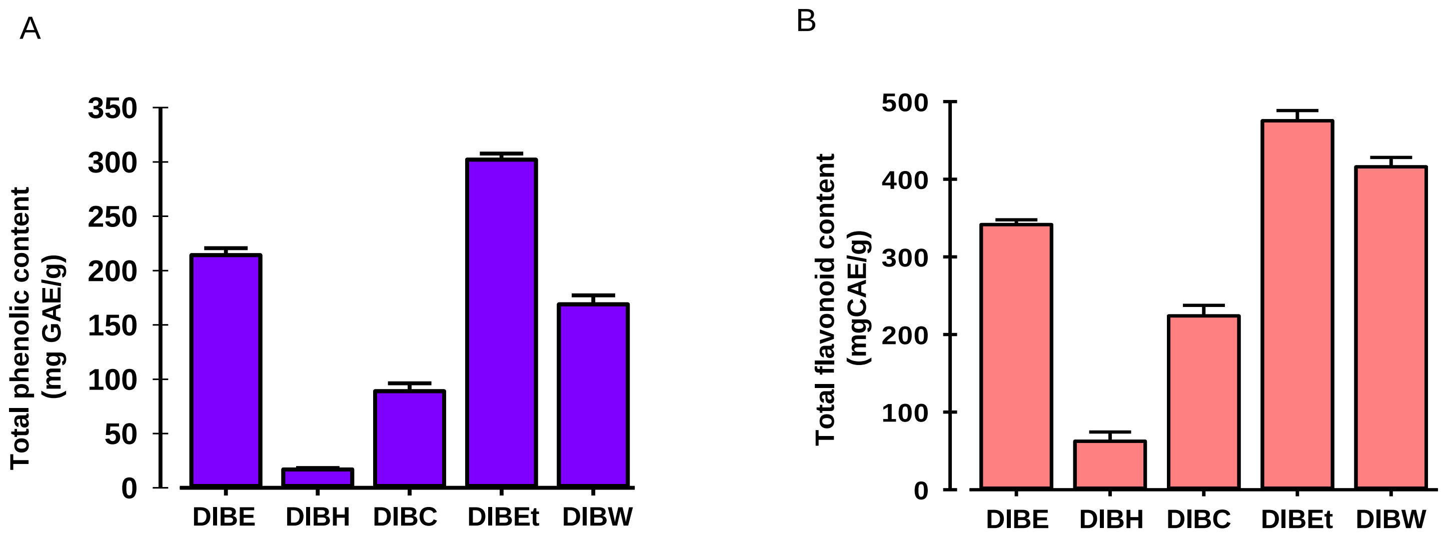

Figure S8: Total phenols (A) and flanovoids (B) content of various organic and aqueous extracts of *D. indica* bark. DIBE: ethanol extract; DIBH: hexane extract; DIBC: chloroform extract; DIBEt: ethylacetate extract and DIBW: aqueous extract of *D. indica* bark

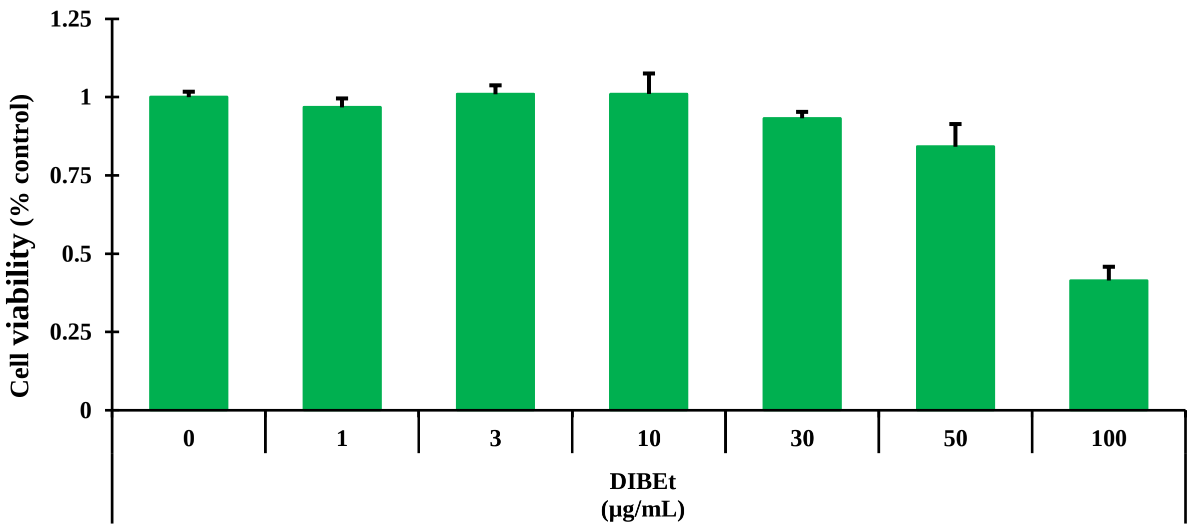

Figure S9: Cell viability of the ethylacetate fraction of *D. indica* bark (DIBEt)

Table S1: List of the primer sets used in this study.

Gene name		Sequences
Sod1	forward	AAGCGGTGAACCAGTTGTGT
	reverse	GCCAATGATGGAATGCTCTC
Gpx1	forward	ACACCGAGATGAACGATCTG
	reverse	ATGTACTTGGGGTCGGTCAT
Catalase	forward	CACCCACGATATCACCAGATAC
	reverse	GAAGACTCCAGAAGTCCCAGAC
Hmox-1	forward	ACGCATATACCCGCTACCTG
	reverse	TCCTCTGTCAGCATCACCTG
Gapdh	Forward	TTGTGATGGGTGTGAACCAC
	reverse	ACACATTGGGGGTAGGAACA

Table S2: List of the primary antibodies used in the study.

Name	Catalog no.	Company	Antigen	Host	Dilutions	Membrane
Anti-SOD1	BS91268	Bioworld Technology, Inc.	SOD1	Rabbit	1:1000	Nitrocellulose membrane
Anti-catalase	BS90194	Bioworld Technology, Inc.	Catalase	Rabbit	1:1000	Nitrocellulose membrane
anti-GPx-1	MB9027	Bioworld Technology, Inc.	GPx-1	Mouse	1:1000	Nitrocellulose membrane
Anti-HO-1	sc-136256	Santa Cruz Biotechnology, Inc.	HO-1	Mouse	1:1000	Nitrocellulose membrane
Anti Nrf2	sc-81342	Santa Cruz Biotechnology, Inc.	Nrf2	Mouse	1:1000	Nitrocellulose membrane
Anti-Lamin B	BS3547	Bioworld Technology, Inc.	Lamin B	Rabbit	1:1000	Nitrocellulose membrane
Anti-p-p38	sc-166182	Santa Cruz Biotechnology, Inc.	p38	Mouse	1:1000	Nitrocellulose membrane
Anti-p38	BS3567	Bioworld Technology, Inc.	p38	Rabbit	1:1000	Nitrocellulose membrane
Anti-p-ERK1/2	sc-7383	Santa Cruz Biotechnology, Inc.	ERK	Mouse	1:1000	Nitrocellulose membrane
Anti-ERK1/2	BS 6472	Bioworld Technology, Inc.	ERK	Rabbit	1:1000	Nitrocellulose membrane
Anti-p-JNK	BS 4322	Bioworld Technology, Inc.	JNK	Rabbit	1:1000	Nitrocellulose membrane
Anti-JNK	sc-7345	Santa Cruz Biotechnology, Inc.	JNK	Mouse	1:1000	Nitrocellulose membrane
Anti- βactin	Sc-47778	Santa Cruz Biotechnology, Inc.	β-actin	Mouse	1:1000	Nitrocellulose membrane
